# Adaptive Segmentation of Streaming Sensor Data on Edge Devices

**DOI:** 10.3390/s21206884

**Published:** 2021-10-17

**Authors:** Roman Dębski, Rafał Dreżewski

**Affiliations:** Institute of Computer Science, AGH University of Science and Technology, Al. Mickiewicza 30, 30-059 Kraków, Poland; drezew@agh.edu.pl

**Keywords:** sensor networks, edge computing, data stream smoothing, data stream compression, cubic splinelet, cubic spline

## Abstract

Sensor data streams often represent signals/trajectories which are twice differentiable (e.g., to give a continuous velocity and acceleration), and this property must be reflected in their segmentation. An adaptive streaming algorithm for this problem is presented. It is based on the greedy look-ahead strategy and is built on the concept of a cubic splinelet. A characteristic feature of the proposed algorithm is the real-time simultaneous segmentation, smoothing, and compression of data streams. The segmentation quality is measured in terms of the signal approximation accuracy and the corresponding compression ratio. The numerical results show the relatively high compression ratios (from 135 to 208, i.e., compressed stream sizes up to 208 times smaller) combined with the approximation errors comparable to those obtained from the state-of-the-art global reference algorithm. The proposed algorithm can be applied to various domains, including online compression and/or smoothing of data streams coming from sensors, real-time IoT analytics, and embedded time-series databases.

## 1. Introduction

Sensor signal chain solutions used to rely totally upon cloud infrastructure whenever high-level data processing was required. In most cases, it was effective because the amounts of data to be transferred were small, and possibly existing real-time constraints were not excessive. For contemporary systems, however, this approach is often not acceptable, since the full bandwidth of sampled data will almost always cause network congestion and/or create a significant bottleneck for the aggregation node (e.g., a wireless gateway).

The obvious solution can be to compress the data before uploading it. To realize this and to address the above issues, the edge computing approach emerged, which can be treated as a decentralized cloud that brings computing power and thus capabilities of data stream pre-processing and compression closer to data sources such as sensors, Internet of Things (IoT) devices and wearable devices [1,2].

Locating computing power closer to data sources is indispensable for some applications requiring almost real-time responses, such as for example autonomous vehicles and e-health. Real-time requirements of such applications cannot be met by the regular cloud in the case of numerous sensors because of high latency and ineffective bandwidth [1,2]. The computing power available in edge devices also opens up new possibilities for advanced data stream pre-processing such as smoothing and/or segmentation.

In many instances, certain properties of the input signal—typically represented as a series of data points obtained by sampling—are known and must be considered during the segmentation of the signal. A common example is the signal smoothness, measured by the differentiability class $C^{k}$, with $C^{2}$ often being the target ($f\in C^{2}$ if it is twice differentiable. For instance, in robotics or control systems to have a smooth movement, the trajectory must be twice differentiable to give a continuous velocity and acceleration.).

With no access to future values, an effective algorithm for the segmentation of streaming data, must be entirely local. Although such algorithms exist (for instance, PLA, PMC-MR, Linear Filter [3]), their outputs are not $C^{2}$-continuous. This also refers to cubic Hermite spline-based solutions (segmentations), which are $C^{1}$-continuous only.

The second group of potential solutions—represented by cubic smoothing splines—gives $C^{2}$-continuous outputs, yet the corresponding algorithms are not local since they require the solution of a system of linear equations whose coefficients depend on the whole data set. To the best of our knowledge, there does not exist a streaming algorithm which combines the above properties, i.e., is local and computes $C^{2}$-segmentations.

**Figure 1:**
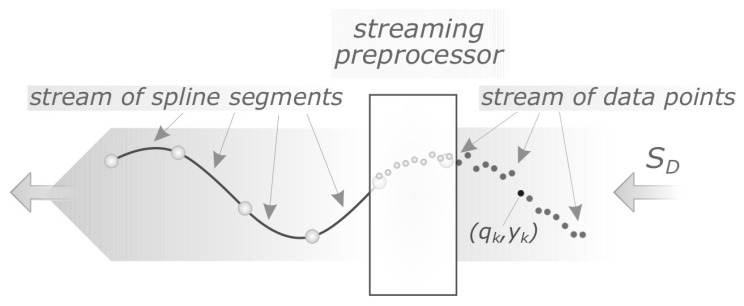
Conceptual diagram of the considered problem: the streaming preprocessor (segmenter) maps a *stream of data points* to a *stream of cubic spline segments*, which form a $C^{2}$-continuous curve.

Our aim is to propose such an algorithm. The presented algorithm is based on the greedy look-ahead strategy and built upon the concept of a cubic splinelet (see Figure 1). One of its key properties is the real-time simultaneous segmentation, smoothing, and compression of noisy data streams. This means it can be applied to various domains including online compression and/or smoothing of streaming data, real-time IoT analytics, and embedded time-series databases.

The main contributions of this paper are the following:

- the cubic splinelet of type $WSSR_{min}$—the special type of splinelet that minimizes the *Weighted Sum of Squared Residuals* (Section 4.1),
- the algorithm for $C^{2}$-continuous $WSSR_{min}$-cubic splinelet-based adaptive segmentation of streaming sensor data (Section 4.2 and Section 4.3),
- numerical results which demonstrate the effectiveness of the algorithm (Section 5).

The remainder of this paper is organized as follows. The next Section 2 contains the related work overview. Following that Section 3, the problem statement is given and then, in Section 4, the proposed solution is described. Next Section 5, the solution is evaluated, and the obtained results are presented and discussed. The last Section 6 contains the conclusion of the study.

## 2. Related Work

Stream computing requires low-latency real-time algorithms that can process massive amounts of data generated by multiple sources at very high speed [4]. Such algorithms should be able to pre-process and analyze on-the-fly high-velocity streams of data coming from sources such as the Internet of Things (IoT) devices, sensor networks, wearable and mobile devices, market data.

The key value of data coming from such sources is their “freshness”, and they should be processed and analyzed as soon as they arrive, which is the key assumption of the big data stream analytics [4]. Such a requirement leads to the need for low-latency real-time algorithms because the batch computing approach, in which data should be stored first before it is processed and analyzed, is not sufficient [4,5,6]. In recent years, the research has mainly been focused on algorithms for real-time analysis of big data streams and there was not much research into the noisy or incomplete streaming data pre-processing phase [4].

Below, the research related to the proposed algorithm characteristic features—the real-time data stream segmentation, smoothing, and compression—is presented. Moreover, the related research works on splines are mentioned and the selected possible application areas for the proposed algorithm are reviewed.

### 2.1. Data Stream Segmentation

Sensors located in IoT devices generate data streams continuously. For some application areas, it is crucial to partition such data into segments to perform successful analysis using advanced algorithms, for example the machine learning ones.

Streaming segmentation of the signal realized on edge devices allows for:

- reconstruction of a sampled noisy signal (to maintain its continuity/smoothness class as a key feature (like non-negativity)) before the network transmission,
- signal compression (in experiments with test signals, we observed the data size reduction from 135 to 208 times, i.e., two orders of magnitude),
- reduction of network traffic (in the entire infrastructure),
- energy savings (in the entire infrastructure—the network communication is energy-intensive, and additionally the signal smoothed on edge devices no longer needs to be pre-processed in the cloud).

Recognition and prediction of human activities by real-time analysis of data streams coming from sensors and actuators is one of the areas of application of data stream segmentation [7]. The problem of automatic segmentation of data stream into activities in real time is a difficult one—there is no general approach for determining the end of the detected activity [7]. Sensor data stream segmentation has been the subject of many research works. Some proposed approaches were based on a time window with fixed length or on a dynamic time window including fixed number of events [8,9]. The other approaches were based on a real-time analysis of temporal information but either very intensive pre-processing was required, or the application was limited (for example to location analysis) [10,11].

An approach for continuous activity recognition based on the real-time sensor data segmentation was proposed in [12]. The proposed method was based on dynamically resized time windows (taking into account temporal sensor data and the state of activity recognition) and the ontology-based activity recognition algorithm. A real-time activity prediction method using automatic data stream segmentation based on Jaro–Winkler distance measurement was proposed in [7].

A method for unsupervised on-the-fly segmentation and classification of the time-series data was proposed in [13]. The approach was based on data density distribution estimation and the data stream was processed incrementally, using fixed amount of resources (memory and CPU). It even worked in real time when the sampling rate of the data stream was on the certain level [13]. A semantic-based approach for real-time separating and segmenting sensor data stream into multiple threads of activities was proposed in [14].

None of the above-mentioned approaches can provide online $C^{2}$-continuous segmentation. This is crucial if we must deal with physical constraints (for example, velocity and acceleration must be continuous).

Our proposed adaptive segmentation of the data stream (sampled signal) takes place in the approximation space of 3rd order splines (which represents the space of twice differentiable functions, i.e., the problem domain). Its result is a stream of segments that represents the reconstructed true signal. A spline constructed in this way recreates the signal taking into account its known class of continuity (smoothness). Therefore, we segment the data stream taking into account the features (continuity/smoothness class) of the processed signal.

### 2.2. Data Stream Smoothing

Advanced driving assistance systems and adaptive cruise control systems require high-accuracy, low-noise (or at least smoothed) data for proper functioning [15]. Data streams of estimated vehicle position can be obtained from different types of sensors: GPS, radar, LiDAR, gyroscopes, accelerometers, wheel speed sensors. Data coming from those sensors can be noisy and inaccurate due to many technical reasons. Such inaccuracies may lead to incorrect absolute positioning, unrealistic kinematics and inconsistent spacing between vehicles [15].

In car-following applications, the Kalman smoothing was used for improving the quality of data coming from one source (GPS) [16] or from multiple sensors [15,17]. The Kalman smoothing approach was also used for improving the vehicle positioning data coming from GPS and internal dead reckoning (gyroscopes, accelerometers, wheel speed) sensors [18].

The method for smoothing the data stream coming from ultrasonic sensors measuring the water level was proposed in [19]. The proposed approach included outlier detection using modified Z-scores based on the median absolute deviation and stream data smoothing based on the exponentially weighted moving average.

A relational database system was extended to include real-time method based on dynamic probabilistic models for filtering and smoothing data streams in [20]. In their approach, the authors used particle filters (a class of sequential Monte Carlo algorithms).

The data stream smoothing methods mentioned above do not include data stream segmentation nor compression. The approach using wavelet-based Kalman data smoothing for processing uncertain oil well-testing data, which included compression, was presented in [21]. However, the backward data smoothing was performed offline. The real-time approach proposed in this paper provides $C^{2}$-continuous segmentation and data smoothing.

Signals of $C^{2}$ continuity, recorded by sensors, constitute a substantial category/class (for example, recorded location of autonomous vehicles, drones or industrial robots). $C^{2}$ continuity (as a measure of smoothness) is a key characteristic of a signal (similar to, for example, monotonicity or non-negativity) and determines the problem domain. It means that the signal can be differentiated twice. For example, it allows, based on the recorded location of the object, the determination of its velocity and acceleration, without the need to additionally register these quantities which, in turn:

- significantly reduces the size of the data needed to be transferred from the edge layer to the cloud,
- reduces delays and speeds up data transmission,
- reduces energy consumption (in the entire infrastructure).

In the case of a noisy signal (a typical case), taking into account the continuity class allows for a more accurate reproduction of the true signal (noise removal). The continuity class is an important element of our knowledge about the signal, which we should not ignore because it reduces the accuracy of the true signal reproduction (the signal cannot represent, for example, the function of the object’s location in time, if it is not twice differentiable—otherwise it would allow the possibility of the operation of infinitely large forces, and we do not have such in nature).

### 2.3. Data Stream Compression

The cloud computing is an indispensable part of the Internet of Things (IoT). However, using it gives us many problems including transmission latency, bandwidth constraints, and high energy consumption [2]. Micro-controller, transceiver, and sensor units are the parts of smart devices that consume most of the energy, and data transmission is the most power-hungry task [22,23]. Energy efficiency can be improved by moving computation tasks from the cloud to edge devices, and by reducing the amount of data transferred from IoT devices to the edge (which additionally conserves the edge devices’ storage space) [1,2,24].

The proposed approaches for data reduction during transmission between IoT devices and edge computing devices were based on adaptive sampling [25,26,27], aggregation and compression [28,29,30,31,32], and Compressive Sensing [33,34,35,36]. The main disadvantage of the above approaches is the low compression ratio of non-stationary data coming from multiple sensors [2].

To address the above issue, in [2] a lightweight version of fast error-bounded lossy compression algorithm [37] was proposed. The authors showed that the proposed approach was able to reduce the amount of data transmitted from the wearable device to the edge device by approximately 103 times, simultaneously not worsening the results of data analytics.

None of the above-mentioned compression algorithms pre-process data into a form that would potentially accelerate the operation of machine learning algorithms on edge devices, for example by segmenting or smoothing the data stream before sending it from sensors or IoT devices.

Our algorithm segments the data stream (sampled signal belonging to the continuity class $C^{2}$) in an online way. Segmentation allows for a significant degree of compression (we have observed the data size reduction of 135 to 208 times) and the $C^{2}$ class of the signal helps in this process since three out of four coefficients of each spline segment (apart from the first one) can be calculated from the continuity conditions. The omission in the segmentation process of the known (in advance—because we know what we are measuring) continuity class of the processed signal could potentially allow for a slightly better compression ratio, but at the cost of the accuracy of signal reproduction (and in many cases it is unacceptable). It is primarily about recreating the qualitative characteristics of the signal (continuity/smoothness class), and less about the accuracy of approximation (quantitative feature, measured, for example, with the mean square error).

### 2.4. Splines

A spline—flexible strip of wood that was used to draw smooth curves—was mentioned for the first time in [38], as indicated in [39]. The new idea of a spline curve represented as piece-wise polynomial curves with certain smoothness properties was proposed in [40]. In this work, mathematical foundations for spline interpolation and approximation were presented. More information on splines can be found for example in the following works [41,42,43,44,45].

An approximation of a linearly varying curvature by three cubic curve segments was proposed in [46]. An online algorithm for the generation of minimum time joint industrial manipulators trajectories, using similar representation of a curve as in [46], was proposed in [47].

The real-time and online applications of interpolating splines were proposed in [48,49,50,51,52,53,54,55]. Among others, the application areas included trajectory generation and planning methods for robotic [53,54] and simulation-based sailboat trajectory optimization was [55].

To the best of our knowledge, there is no online algorithm, which can compute $C^{2}$-continuous segmentations. The approach proposed in this paper can perform online $C^{2}$-continuous cubic splinelet-based adaptive segmentation. It can process data streams in real time. It can also be applied offline when dealing with huge amounts of data, which cannot be processes by traditional algorithms due to memory limitations.

### 2.5. Possible Application Areas

The application areas, for which there is a need for on-the-fly algorithms allowing for data stream segmentation, smoothing, and compression include, but are not limited to, IoT devices, sensor networks, edge computing, and autonomous vehicles (cars, robots, drones). The need for real-time pre-processing of big data streams coming from multiple sensors results from data noise, bandwidth limitations and energy efficiency requirements. Below, the selected research in three areas (sensor networks, Unmanned Aerial Vehicles teams and robot teams), in which the proposed algorithm could be applied, is presented.

Weather prediction generally requires expensive weather stations and supercomputers for computations. An alternative can be the approach using Distributed Sensor Network for collecting data and performing weather prediction computations [56].

Such an approach requires real-time pre-processing, segmentation, smoothing, and compression of data streams because the used weather stations continuously communicate with each other and exchange large amounts of data coming from sensors to compute the predictions. The approach proposed in this paper meets all the requirements to be used in this area of applications—it allows for online $C^{2}$-continuous cubic splinelet-based adaptive segmentation, compression, and smoothing of noisy data streams.

Using multiple Unmanned Aerial Vehicles (UAVs) for surveillance, environmental monitoring, and rescue operations has become an increasingly popular research topic in the recent years [57,58]. Tracking single or multiple moving ground targets requires continuously updated and accurate data about their position. The accuracy of data coming from UAV’s sensors is crucial for that task. However, data coming from sensors such as GPS, radar, LiDAR, gyroscopes, and accelerometers can be noisy and inaccurate due to many technical reasons. Using multiple coordinated UAVs allows for combining data coming from their sensors and thus using more accurate information about the current target(s) position [57]. Furthermore, the navigation and coordination of the group of UAVs will require real-time continuous exchange of large amounts of data coming from each unit sensors [59].

Using a real-time algorithm that can segment, smooth and compress data streams coming from each UAV’s sensors will be crucial for successfully navigating and coordinating a whole team. The online algorithm proposed in this paper not only computes $C^{2}$-segmentations, but also smooths and compress data in real time, which makes it fully applicable in such a domain as UAVs’ sensors data analytics. The online $C^{2}$-continuous segmentation is crucial for UAVs because, for example, the approximated trajectory of a vehicle must be twice differentiable to give a continuous velocity and acceleration.

The research on multi-robot systems (MRS) gained importance and developed significantly in the recent years. Some of the most important research problems in MRS domain include communication mechanisms, planning and coordination strategies, and decision-making algorithms [60]. Research issues related to team coordination [61], sharing data, intelligence, and resources between many robots [62] are of great importance. The effective communication between many robots in the case of limited bandwidth resulting from environmental conditions (for example underwater environment), in which teams of robots are operating, is also the subject of intensive research [63]).

As in the case of drone teams, also in the case of robot teams coordinating their actions and sharing data, the essential issue is to deal in real time with data streams, which additionally can be noisy and incomplete. In such a case, using a method of segmenting, smoothing and compressing data in real time is crucial. The proposed approach not only does this, but also provides $C^{2}$-segmentations, which is of crucial importance when we use the data to plan the trajectories for robots. In such a case the trajectory must be twice differentiable to give a continuous velocity and acceleration.

## 3. Problem Formulation

Consider a stream of sensor data points, $S_{D}=\left(D_{0},D_{1},D_{2},\ldots \right)$, that arrive (or are accessed) sequentially, and describe an underlying signal f(q), q∈R (note that in the subsequent formulae the *q* stands for any independent variable, typically it will refer to time (*t*)), where:

(1) $$D_{k}=\left(q_{k},f\left(q_{k}\right)\right)=\left(q_{k},y_{k}\right).$$

This stream in a general case is “noisy”, i.e.,

(2) $$f\left(q_{k}\right)=g\left(q_{k}\right)+\epsilon_{k},\;k=0,1,2\ldots$$

where g(·) is the *true signal* and $\epsilon ∼N(\mu ,\;\sigma^{2})$, i.e., it is Gaussian noise. This model is shown in Figure 1.

**Problem** **statement.** *Given a stream of data points $S_{D}=\left(D_{0},D_{1},D_{2},\ldots \right)$, where $D_{k}=\left(q_{k},y_{k}\right)$ with $y_{k}=g\left(q_{k}\right)+\epsilon_{k},\;k=0,1,2\ldots$, find the $C^{2}$-continuous cubic spline whose segments correspond—in the space generated by the user-defined segment length adaptation strategy (δ)—to the optimal segmentation of $S_{D}$, with regard to the reconstruction of the original signal (g).*

**Remark** **1.** *The adaptation strategy, δ, usually depends on the target platform capabilities (e.g., the available memory), and on the required accuracy of the solution. In specific cases, it can be very sophisticated, e.g., Machine Learning (ML)-based.*

## 4. Proposed Solution

The streaming algorithm we propose is based upon the concept of a *cubic splinelet*—a *local* building block of an “on the fly” constructed *global* cubic spline, which is by definition $C^{2}$-continuous (see Section 4.1 and [64]). This local, three-segment building block introduces a look-ahead capability to the algorithm, which—because of its online characteristic—must be *greedy*. Indeed, we can construct the global cubic spline using only the first segment of each splinelet, while the remaining two—treated as a “look-ahead” part—can be dropped (see Algorithm 1).

The key elements of the proposed algorithm (including its pseudo-code) are given in the following three subsections.

### 4.1. Cubic Splinelet of Type $WSSR_{min}$—The Solution Building Block

Without loss of generality, we can consider the problem in the following local frame:

(3) $$\left(x,y\right)=\left(q-q_{0},y\right)$$

which means that an interval $\left[q_{A},q_{D}\right]$, given in the global frame, Oqy, is shifted in *q*-direction by the *offset*, $q_{0}$:

(4) $$\left[q_{A},q_{D}\right]\overset{\mathrm{shift}\mathrm{by}q_{0}}{\to }\left[x_{A},x_{D}\right]=\left[q_{A}-q_{0},q_{D}-q_{0}\right]$$

In a special case, when $q_{0}=q_{A}$, we get:

(5) $$\left[q_{A},q_{D}\right]\overset{\mathrm{shift}\mathrm{by}q_{A}}{\to }\left[x_{A},x_{D}\right]=\left[0,q_{D}-q_{A}\right]$$

*Note*: this local frame, Oxy, will be used in the following paragraphs.

**Definition** **1.** *A cubic splinelet of type $WSSR_{min}$ is a three-segment piece-wise cubic function defined in the local frame (when $q_{0}=q_{A}$) as (*[64]*):*

(6) $$s\left(x\right)=\left\{\begin{array}{cc} s^{\left(1\right)}\left(x\right) & 0\leq x\leq x_{B}\\ s^{\left(2\right)}\left(x\right) & x_{B}\leq x\leq x_{C}\\ s^{\left(3\right)}\left(x\right) & x_{C}\leq x\leq x_{D} \end{array}\right.$$

*where:*

(7) $$s^{\left(i\right)}\left(x\right)=\sum_{j=1}^{4}a_{j}^{\left(i\right)}x^{4-j},\;i=1,2,3$$

*and with the following properties:*

- *s(x) is $C^{2}$-continuous on the interval $I_{s}=\left[0,x_{D}\right]$,*
- *s(x) has the following boundary conditions:*
(8) $$x_{A}=0:\left\{\begin{array}{c} s\left(x_{A}\right)=s_{A}\\ s^{\prime }\left(x_{A}\right)=s_{A}^{\prime }\\ s^{\prime \prime }\left(x_{A}\right)=s_{A}^{\prime \prime } \end{array}\right.$$
- *minimizes the Weighted Sum of Squared Residuals, i.e.,*
(9) $$\mathrm{WSSR}=\sum_{i=1}^{3}\sum_{x_{k}\in I_{s}^{\left(i\right)}}w_{i}\left(x_{k}\right){\left[y_{k}-s^{\left(i\right)}\left(x_{k}\right)\right]}^{2}\to min$$
  *where: $I_{s}^{\left(1\right)}=\left[0,x_{B}\right)$, $I_{s}^{\left(2\right)}=\left[x_{B},x_{C}\right)$ and $I_{s}^{\left(3\right)}=\left[x_{C},x_{D}\right]$.*

To find the splinelet corresponding to Equation (9), we first note that each coefficient in Equation (7) can be expressed in the following way:

(10) $$a_{j}^{\left(i\right)}=A_{j1}^{\left(i\right)}s_{D}+A_{j2}^{\left(i\right)}s_{D}^{\prime }+A_{j3}^{\left(i\right)}s_{D}^{\prime \prime }+A_{j4}^{\left(i\right)}=\sum_{l=1}^{4}A_{jl}^{\left(i\right)}\alpha_{l}$$

where: i=1,2,3, j=1,2,3,4, and $\left(\alpha_{1},\alpha_{2},\alpha_{3},\alpha_{4}\right)=\left(s_{D},s_{D}^{\prime },s_{D}^{\prime \prime },1\right)$. Equation (9) can be now restated as the following *parametric optimization problem*:

(11) $$\mathrm{WSSR}=J\left(\alpha_{1},\alpha_{2},\alpha_{3}\right)=\sum_{i=1}^{3}\sum_{x_{k}\in I_{s}^{\left(i\right)}}w_{i}\left(x_{k}\right){\left[y_{k}-\sum_{j=1}^{4}\sum_{l=1}^{4}A_{jl}^{\left(i\right)}\alpha_{l}x_{k}^{4-j}\right]}^{2}\to min$$

or, in another notation:

(12) $$\underset{\alpha_{1},\alpha_{2},\alpha_{3}}{\arg\;\min}\;\sum_{i=1}^{3}\sum_{x_{k}\in I_{s}^{\left(i\right)}}w_{i}\left(x_{k}\right){\left[y_{k}-\sum_{j=1}^{4}\sum_{l=1}^{4}A_{jl}^{\left(i\right)}\alpha_{l}x_{k}^{4-j}\right]}^{2}.$$

Next, we compute:

(13) $$\left\{\begin{array}{c} \frac{\partial J}{\partial \alpha_{1}}\left(\alpha_{1},\alpha_{2},\alpha_{3}\right)=0\\ \frac{\partial J}{\partial \alpha_{2}}\left(\alpha_{1},\alpha_{2},\alpha_{3}\right)=0\\ \frac{\partial J}{\partial \alpha_{3}}\left(\alpha_{1},\alpha_{2},\alpha_{3}\right)=0 \end{array}\right.$$

which leads to a system of three linear equations (since $J(\alpha_{1},\alpha_{2},\alpha_{3})$ is linear with respect to $\alpha_{i}$, i=1,2,3):

(14) $$\sum_{j=1}^{3}B_{ij}\alpha_{j}=C_{i},\;i=1,2,3$$

whose solution (i.e., the optimal values of $\alpha_{i}$, i=1,2,3) we substitute into Equation (10), and obtain the coefficients of the corresponding splinelet.

**Remark** **2.** *Having set $w_{i}\left(x\right)$, i=1,2,3, and $x_{A}$, $x_{B}$, $x_{C}$, $x_{D}$, we can find the closed-form solution (compare *[64]*) for $a_{j}^{\left(i\right)}$, i=1,2,3, j=1,2,3,4.*

### 4.2. Segmentation Heuristic Overview

The cubic splinelet defined in the previous section gives the locally optimal *approximant* in the given interval $\left[0,x_{D}\right]$. However, the following questions remain unanswered:

- What should be the value of $x_{D}$ that corresponds to the locally optimal stream segmentation/partitioning?
- What should be the search space for this optimization task?

The search space (interval) can be straightforwardly derived from the selected spline adaptation strategy. It may be as simple as:

(15) $$Dom\left(x_{D}^{\left(i\right)}\right)=\left[x_{D_{S}}^{\left(i\right)},x_{D_{E}}^{\left(i\right)}\right]=\left[\frac{1}{2},2\right]\;x_{D}^{\left(i-1\right)}$$

The best $x_{D}$ can be then computed using an iterative improvement method. At each of its steps:

1. the search interval is divided into predefined number of sub-intervals,
2. they are then evaluated (see Equation (16) below) by sampling and interpolating (*note*: this step can be accelerated by memoization/caching),
3. the best sub-interval becomes the new search interval.

The fitness (objective) function, ϕ, used in the above search algorithm is defined in the following way:

(16) $$\phi \left(s;\Delta R_{adj}^{2},{\bar{L}}_{1}{}_{min},{\bar{L}}_{1}{}_{max}\right)=\phi_{1}\left(s\right)+\Delta R_{adj}^{2}\;\phi_{2}\left(s;{\bar{L}}_{1}{}_{min},{\bar{L}}_{1}{}_{max}\right)$$

where:

(17) $$\phi_{1}\left(s\right)=R_{adj}^{2}\left(s\right)=1-\frac{\sum_{k=1}^{n}{\left[y_{k}-s\left(x_{k}\right)\right]}^{2}}{\sum_{k=1}^{n}{\left(y_{k}-\bar{y}\right)}^{2}}\frac{n-1}{n-4}$$

(18) $$\phi_{2}\left(s;{\bar{L}}_{1}{}_{min},{\bar{L}}_{1}{}_{max}\right)=\left\{\begin{array}{cc} \frac{{\bar{L}}_{1}{}_{max}-{\bar{L}}_{1}\left(s\right)}{{\bar{L}}_{1}{}_{max}-{\bar{L}}_{1}{}_{min}}, & {\bar{L}}_{1}{}_{max}-{\bar{L}}_{1}{}_{min}>0\\ 1, & \mathrm{otherwise} \end{array}\right.$$

and:

(19) $$\bar{y}=\frac{1}{n}\sum_{k=1}^{n}y_{k}$$

(20) $$\Delta R_{adj}^{2}=\max_{s\in \sum_{s}}R_{adj}^{2}\left(s\right)-\min_{s\in \sum_{s}}R_{adj}^{2}\left(s\right)$$

(21) $${\bar{L}}_{1}{}_{max}=\max_{s\in \sum_{s}}\mathrm{MAE}\left(s\right)=\max_{s\in \sum_{s}}\frac{1}{n}\sum_{k=1}^{n}\left|y_{k}-s\left(x_{k}\right)\right|$$

(22) $${\bar{L}}_{1}{}_{min}=\min_{s\in \sum_{s}}\mathrm{MAE}\left(s\right)=\min_{s\in \sum_{s}}\frac{1}{n}\sum_{k=1}^{n}\left|y_{k}-s\left(x_{k}\right)\right|$$

with $R_{adj}^{2}$ being the *Adjusted Coefficient of Determination*, *MAE*—the *Mean Absolute Error*, and $\sum_{s}$—a set of candidate splinelets (corresponding to different values of $x_{D}$).

### 4.3. The Algorithm

Algorithm 1 presents a high-level view of the whole computational process (see also Appendix A). The *sliding window*-based streaming segmentation that we propose is clearly reflected in its structure. It is also worth noting that:

- the sliding window buffer size, *h*, can be either fixed upfront (e.g., depending on the input signal characteristics and/or real-time constraints), or constantly adapted (e.g., using ML algorithms); a good strategy for the first approach is to use the value of *h* corresponding to the maximum acceptable buffering delay (latency),
- to increase the readability of the pseudo-code, checking for exceptional/corner cases (e.g., too few data points at the end of the sliding window buffer to build one more segment) was omitted in some places,
- the algorithm presents one possible way of handling the end of the stream ($s_{Best}^{\left(3\right)}$, was computed with no looking-ahead); again, if necessary, this computation can be more sophisticated (e.g., the stream can be “artificially” extended),
- the number of sub-intervals that a given interval is divided into can be either fixed upfront or variable (e.g., simple dependence on the length of the interval, or ML-based).

**Remark** **3.** *The streaming characteristic of the algorithm means that it requires O(n) time and O(h) space, where n stands for the length of $S_{in}$ (potentially n→∞). See also Appendix B.*

**Algorithm 1.** Adaptive segmentation of streaming data (see also Appendix A)

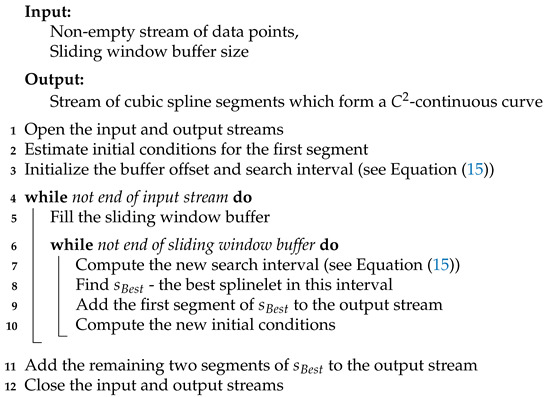



## 5. Results and Discussion

To evaluate the proposed algorithm, a series of numerical experiments was carried out, mostly in the form of a comparative analysis. As a point of reference, the results obtained from R function smooth.spline (accessed on 15 July 2021) were used. It is worth noting that this function—an example of state-of-the-art solutions—*is global* (i.e., the whole stream must be given as its input).

A summary of the evaluation process used is given in Section 5.1 and the results of the experiments are presented in Section 5.2 and Section 5.3.

### 5.1. Evaluation Process Overview

The key aspects of the evaluation process—*test streams*, *algorithm performance descriptors*, and *the reference function (algorithm) used*—are briefly described in this section.

#### 5.1.1. Test Streams

**Figure 2:**
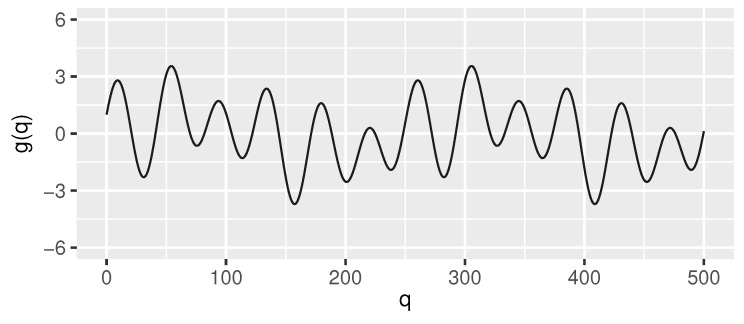
Signal *g* (Equation (2), for readability shown only in the interval [0,500]) used—after discretization and adding Gaussian noise—to generate the test streams used in the evaluation of the proposed algorithm.

**Figure 3:**
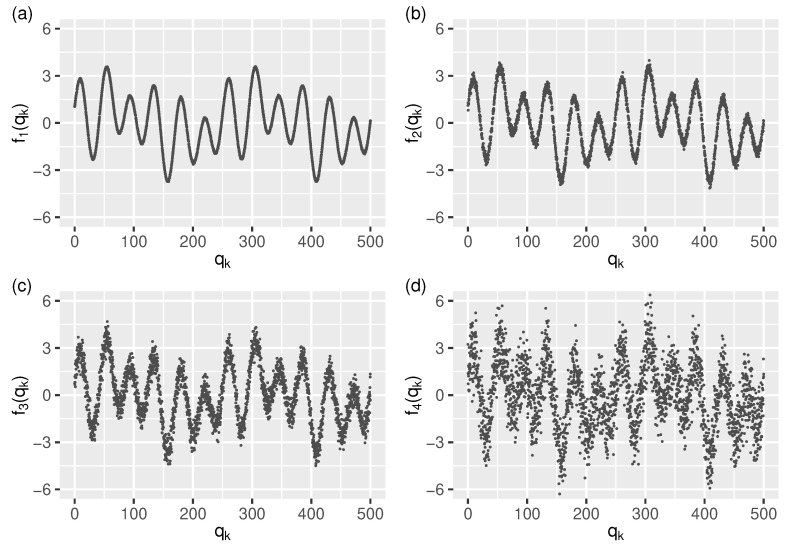
The test streams: (**a**–**d**) $f_{1}$–$f_{4}$ (for readability shown only in the interval [0,500]) generated from signal *g* using four levels of Gaussian noise (see Equation (27)).

The test data sets were generated using the following function *g* (its graph in the interval [0, 500] is shown in Figure 2) as the “true signal”:

(23) g(q)=sin(π/2−0.1q)+sin(0.025q)+2sin(0.15q)

to which four levels of Gaussian noise was added, resulting in the following four test streams (see Figure 3):

(24) $$\left\{\begin{array}{c} f_{1}\left(q_{k}\right)=g\left(q_{k}\right)+\epsilon_{1_{k}}\\ f_{2}\left(q_{k}\right)=g\left(q_{k}\right)+\epsilon_{2_{k}}\\ f_{3}\left(q_{k}\right)=g\left(q_{k}\right)+\epsilon_{3_{k}}\\ f_{4}\left(q_{k}\right)=g\left(q_{k}\right)+\epsilon_{4_{k}} \end{array}\right.$$

where:

(25) $$q_{k}=0.05\;k,k=0,1,\ldots ,\;10^{6}$$

(26) $$\epsilon_{i_{k}}=\mathrm{first}\;\left[\;e\;|\;e\leftarrow N\left(\mu =0,\sigma =\epsilon_{i_{max}}/3\right),\;e<\epsilon_{i_{max}}\;\right]$$

i.e., the first *e*, such that: e∼N(μ,σ) and $e<\epsilon_{i_{max}}$, and

(27) $$\left[\;\epsilon_{1_{max}},\epsilon_{2_{max}},\epsilon_{3_{max}},\epsilon_{4_{max}}\;\right]=\left[\;0.1,0.5,1.5,3.5\;\right]$$

#### 5.1.2. Performance Descriptors

Each of the solutions, *s*, was evaluated using the following measures:

- *Mean Absolute Error*:
  (28) $$\mathrm{MAE}\left(s,f\right)=\frac{1}{n}\sum_{k=1}^{n}\left|s\left(x_{k}\right)-f\left(x_{k}\right)\right|$$
- *Root Mean Squared Error*:
  (29) $$\mathrm{RMSE}\left(s,f\right)={\left\{\frac{1}{n}\sum_{k=1}^{n}{\left[s\left(x_{k}\right)-f\left(x_{k}\right)\right]}^{2}\right\}}^{1/2}$$
- *Normalized Root Squared Error*:
  (30) $$\mathrm{NRSE}\left(s,f\right)={\left\{\frac{\sum_{k=1}^{n}{\left[s\left(x_{k}\right)-f\left(x_{k}\right)\right]}^{2}}{\sum_{k=1}^{n}{\left[f(x_{k})\right]}^{2}}\right\}}^{1/2}$$
- *Mean Absolute Error Quotient (local-to-global algorithm ratio 1)*:
  (31) $$Q_{\mathrm{MAE}}\left(s,s_{R}\right){|}_{f}=\frac{\mathrm{MAE}(s,f)}{\mathrm{MAE}(s_{R},f)}$$
- *Root Mean Squared Error Quotient (local-to-global algorithm ratio 2)*:
  (32) $$Q_{\mathrm{RMSE}}\left(s,s_{R}\right){|}_{f}=\frac{\mathrm{RMSE}(s,f)}{\mathrm{RMSE}(s_{R},f)}$$
- *Compression Ratio*:
  (33) $$\mathrm{CR}\left(s,f\right)=\frac{\mathrm{uncompressed}\text{-}\mathrm{size}(f)}{\mathrm{compressed}\text{-}\mathrm{size}(f)}=\frac{\mathrm{size}(f)}{\mathrm{size}(s)}=\frac{\mathrm{length}(S_{in})}{2+\mathrm{length}(S_{out})}$$
  *Note*: due to $C^{2}$-continuity of cubic splines we need only $\left\{4+\left[\mathrm{length}\left(S_{out}\right)-1\right]\right\}+\left\{\mathrm{length}\left(S_{out}\right)+1\right\}=2\;\left[2+\mathrm{length}\left(S_{out}\right)\right]$ values.
- *Absolute Error (function)*:
  (34) AE(x;s,f)=|s(x)−f(x)|
- *Squared Error (function)*:
  (35) $$\mathrm{SQE}\left(x;s,f\right)={\left[s(x)-f(x)\right]}^{2}$$

**Remark** **4.** *The above set covers local (AE and SQE), global (MAE, RMSE, and NRSE), and competitive ($Q_{\mathrm{RMSE}}$, $Q_{\mathrm{RMSE}}$, and CR) performance descriptors.*

#### 5.1.3. Reference Algorithm and Its Limitations

Remember that an online (local, streaming) algorithm is one that can process its input piece-by-piece in a serial fashion without having the entire input available from the beginning (as is the case for offline/global algorithms). As a result, it might make “decisions” that later turn out not to be optimal. Consequently, a *local algorithm cannot outperform its global (optimal) counterpart*. To compare these two, a “local-to-global algorithm ratio” is often used.

Unfortunately, this approach cannot be directly applied to the problem under consideration (i.e., adaptive segmentation of streaming data with the use of $C^{2}$-continuous cubic splines) because *there is no other algorithm to compare it with*. With this in mind, we can assume that the stream is finite and then use an existing cubic spline-based approximator as a (global) point of reference. An example of such an approximator is the R language smoothing spline function smooth.spline (accessed on 15 July 2021).

**Figure 4:**
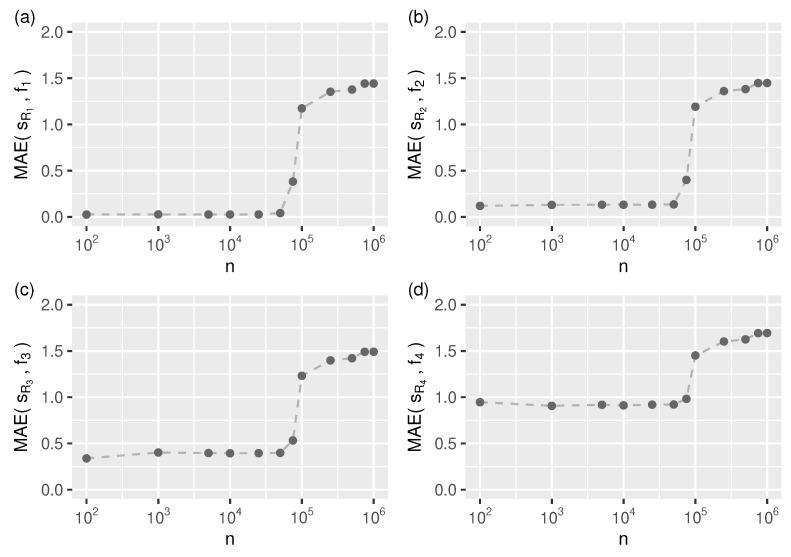
Auto-segmentation related limitations of the reference algorithm: approximation mean absolute error (Equation (28)) as a function of input stream length (*n*) for all test streams, (**a**–**d**) $f_{1}$–$f_{4}$. For $n>6\times 10^{4}$ one needs to specify the number of spline segments (knots) manually.

It turns out, however, that this is still not a solution because from the automatic segmentation point of view, this reference function (algorithm) does not handle data streams longer than about 6% of the length of the test streams (as shown in Figure 4).

**Figure 5:**
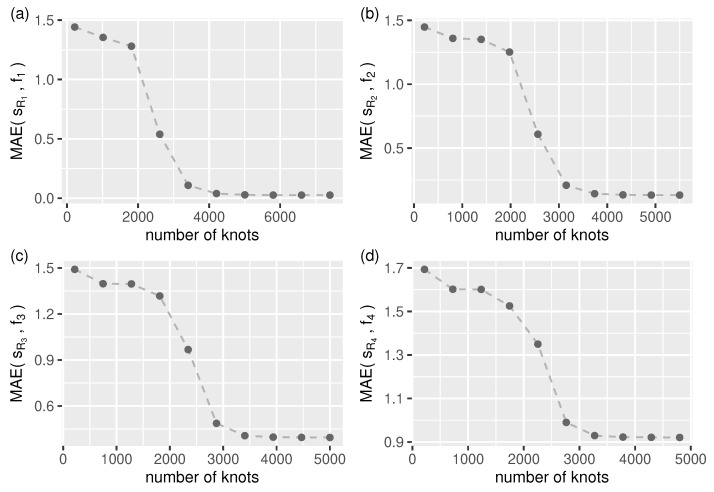
Approximation mean absolute error (Equation (28)) as a function of smooth.spline number of knots (segments) for all test streams, (**a**–**d**) $f_{1}$–$f_{4}$ (in all cases: $n=10^{6}$).

For longer streams, we need to specify the number of smoothing spline segments (knots) manually. As shown in Figure 5, we can expect accurate approximations for all test streams when using more than $4\times 10^{3}$ knots.

**Remark** **5.** *In the evaluation process used, the number of knots for function smooth.spline was set to be the same as that found by the splinelet-based segmentation algorithm (which in all cases was more than $4\times 10^{3}$).*

### 5.2. Evaluation Results: Approximation Errors and Compression Ratio

Given a signal, the quality of its splinelet-based approximation—measured in terms of absolute and quadratic errors (see Section 5.1.2)—is the key performance indicator of the corresponding segmentation which, in turn, is strongly related to the signal compression ratio. The corresponding evaluation results are presented in Table 1 and in Figure 5 and Figure 7.

The values of error quotients QMAE and QRMSE (Table 1) show that the splinelet-based solutions, despite being completely local, in most cases are almost as good as their global (smoothing spline-based) correspondents.

**Figure 6:**
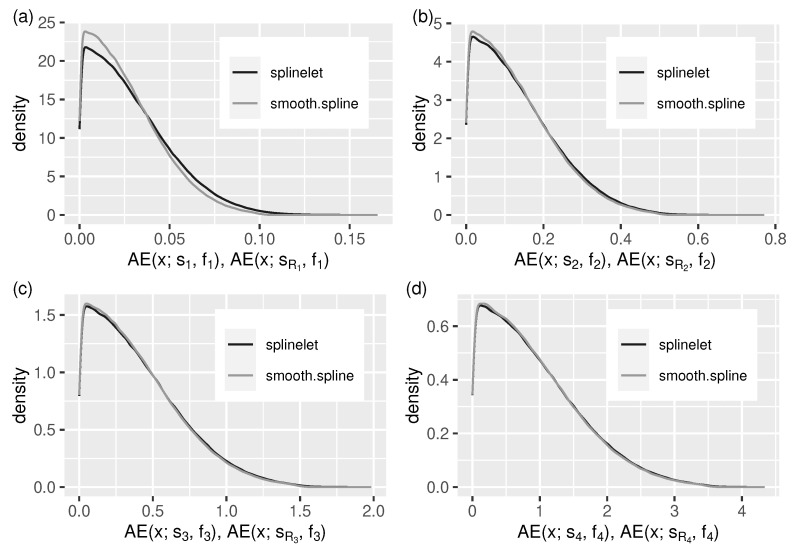
Cubic splinelet generated spline vs. smoothing spline: distribution of *absolute* approximation errors (in the form of a density function) for all test streams, (**a**–**d**) $f_{1}$–$f_{4}$.

**Figure 7:**
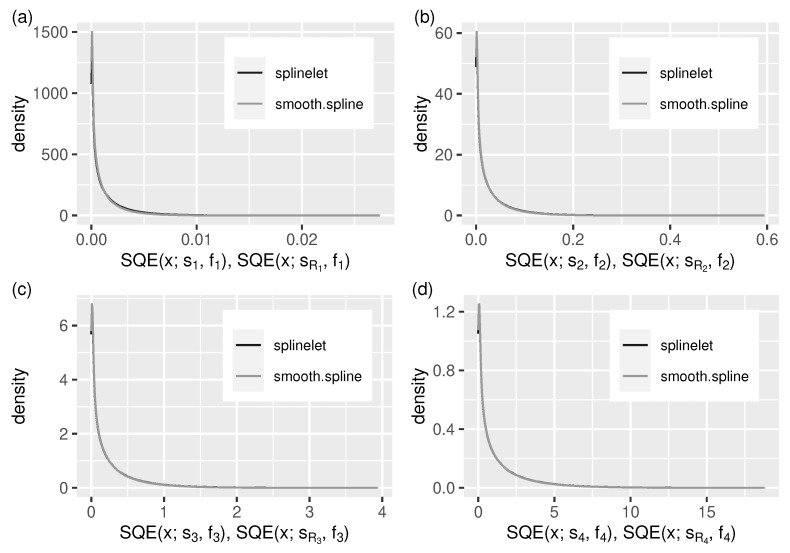
As in Figure 6, but for the *squared* approximation errors, SQE. (**a**–**d**) $f_{1}$–$f_{4}$.

The same can be observed in Figure 6 and Figure 7. They provide additional insight into the splinelet-based approximation. These supplement the integral measure view—given by error quotients—with error distributions. Almost identical shapes of density lines corresponding to the two compared solutions confirm the high quality of splinelet-based solution. In this context, the values of compression ratios, CR(s,t), given in Table 1 can be considered high (from 135 to 208, meaning that the compressed stream sizes are up to 208 times smaller).

### 5.3. Evaluation Results: Segment Length Auto-Adaptation

Since the spline segment length auto-adaptation mechanism determines the search space at each segmentation step, it has significant impact on the algorithm’s overall performance. Not only does this refer to the approximation quality (discussed in Section 5.2), but also—probably even more importantly—to the algorithm’s stability, which becomes essential in the context of the $C^{2}$-continuous streaming approximation.

**Figure 8:**
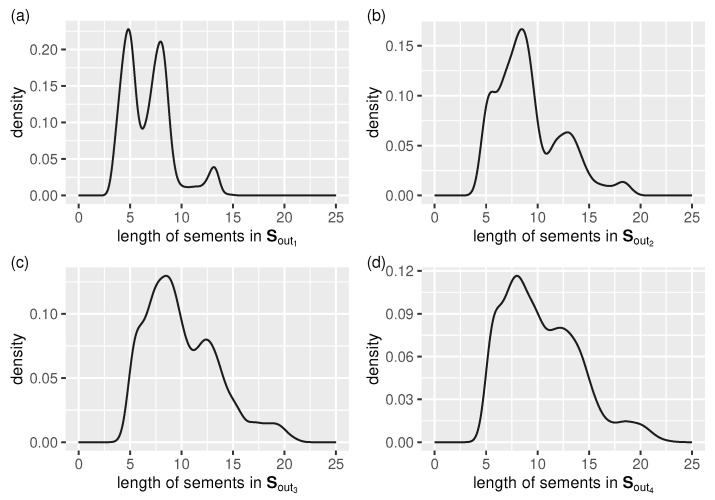
Output stream, $S_{out}$, segment length auto-adaptation: distribution of cubic-splinelet-segment lengths (in the form of a density function) for all test streams, (**a**–**d**) $f_{1}$–$f_{4}$.

Figure 8 shows concisely the spline segment auto-adaptation related results in the form of a segment length distribution for each tested data stream.

We can see that:

- in all cases the dominating segment lengths (remember that the test streams differ only in their signal-to-noise ratios—see Equations (23) and (26)) belong to the interval [5,10],
- the lower the noise level, the more distinct the three existing maxima of the density function become (they correspond to the main “building blocks” used by the segmentation algorithm to restore the true signal, which is periodic),
- the higher the noise level, the closer to uniform the segment length distribution becomes, and the longer the segments are (because of a higher error tolerance).

**Remark** **6.** *Although simple (see Equation (15)), the segment length auto-adaptation mechanism proved effective.*

## 6. Conclusions

It has been shown that the $C^{2}$-continuous cubic splinelet-based adaptive segmentation of streaming data—despite its local/online character—is not only possible but also can be effective. The key element in achieving this was to base the algorithm on the greedy look-ahead strategy based on the concept of a cubic splinelet—a building block for $C^{2}$-continuous cubic splines. A characteristic feature of the proposed algorithm is the simultaneous segmentation, smoothing, and compression of data streams from sensors being performed in real time.

The segmentation quality has been measured in terms of the signal approximation accuracy and the corresponding compression ratio. The numerical results show the relatively high compression ratios (from 135 to 208, see Table 1) combined with the approximation errors comparable to these obtained from the (global) reference algorithm (see Figure 6 and Figure 7).

The proposed algorithm can be applied to various domains, including online compression and/or smoothing of streaming data coming from IoT devices, sensor networks, and sensors located in autonomous vehicles (cars, drones) and robots. The possible application areas also include real-time IoT analytics, and embedded time-series databases. Further exploration of this idea could be the first possible future research direction. Another could be related to more advanced auto-adaptation mechanisms of the search space.

## Figures and Tables

**Table 1:** Performance comparative analysis (see Section 5.1.2): cubic splinelet generated spline (denoted as *s*) vs. smoothing spline (denoted as $s_{R}$) for all test streams $f_{i}$, where i=1,2,3,4.

f	MAE(s,f)	RMSE(s,f)	NRSE(s,f)	QMAE(s,$s_{R}$)	QRMSE(s,$s_{R}$)	CR(s,f)
$f_{1}$	0.029	0.037	0.021	1.110	1.243	135
$f_{2}$	0.136	0.170	0.098	1.033	1.070	183
$f_{3}$	0.402	0.502	0.279	1.019	1.040	201
$f_{4}$	0.933	1.165	0.560	1.013	1.028	208

**Figure A1:**
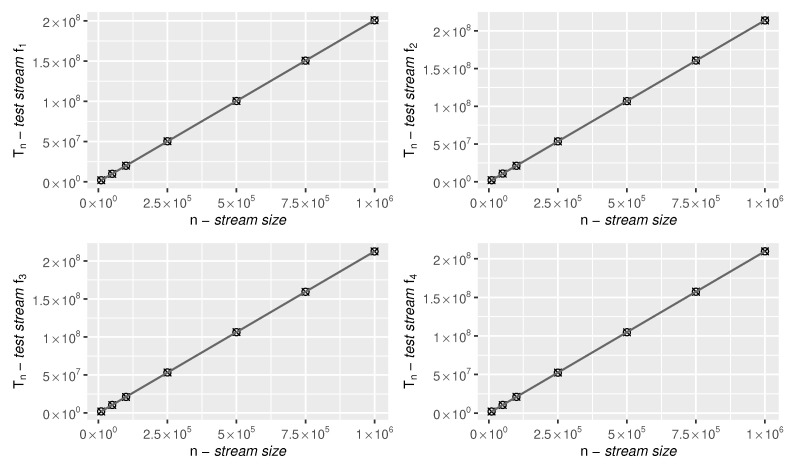
As Table A1, but in graphical form.

## Appendix A. Pseudo-Code of the Proposed Algorithm (Full Version)

**Algorithm A1.** Adaptive segmentation of streaming data (*full version*).

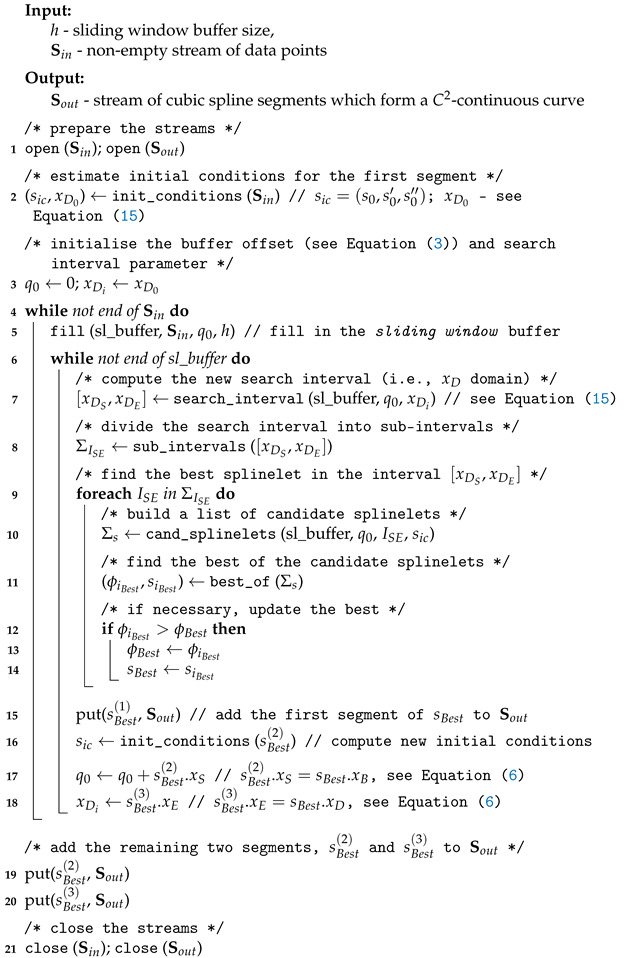

## Appendix B. Experimental Verification of the Algorithm (Linear) Time Complexity

**Table A1:** The algorithm time complexity analysis: $T_{n}$—measured in terms of the number of *representative operations* needed to solve Equation (14)—for different sizes (*n*) of the test streams ($f_{1}$, $f_{2}$, $f_{3}$, and $f_{4}$).

$n\times 10^{3}$	$T_{n}$ for $f_{1}$	$T_{n}$ for $f_{2}$	$T_{n}$ for $f_{3}$	$T_{n}$ for $f_{4}$
10	1,943,679	2,046,967	2,025,182	2,033,942
50	9,860,670	10,791,466	10,472,338	10,535,740
100	19,933,179	21,144,273	21,093,559	21,050,965
250	50,373,800	53,515,682	53,311,644	52,439,474
500	100,428,803	106,829,071	106,302,366	104,768,087
750	150,522,546	160,678,013	159,405,423	157,300,544
1000	200,772,538	213,827,730	212,596,453	209,684,704

**Table A2:** The algorithm time complexity analysis: linear regression ($T_{n}=A\;n+b$).

Test Stream	*A* in $T_{n}=A\;n+b$	Pearson’s $r(n,T_{n})$	$R^{2}$	F Statistics	*p*-Value
$f_{1}$	200.8676	0.9999989	0.9999978	2,228,045	$2.561486\times 10^{-15}$
$f_{2}$	214.0141	0.9999981	0.9999963	1,343,737	$1.239466\times 10^{-15}$
$f_{3}$	212.7101	0.9999992	0.9999983	2,978,702	$1.239466\times 10^{-15}$
$f_{4}$	209.6683	0.9999997	0.9999995	9,631,370	$6.593002\times 10^{-17}$
